# Metabolic Plasticity Versus Conservative Strategies: How Nitrogen Form and Foliar Proline Modulate Genotypic Heat Stress Responses in Tomato

**DOI:** 10.3390/plants15131993

**Published:** 2026-06-27

**Authors:** Yamara González Barrios, María Carmen Piñero, Ginés Otálora, Jacinta Collado-González, Aitziber Calleja Satrustegui, Idoia Ariz, Francisco M. del Amor

**Affiliations:** 1 Department of Crop Production and Agri-Technology, Murcia Institute of Agri-Food Research and Development (IMIDA), 30150 Murcia, Spain; mariac.pinero2@carm.es (M.C.P.); gines.oralora@carm.es (G.O.); jacinta.collado@carm.es (J.C.-G.); 2 Campues Arrosadia, Public University of Navarre (UPNa), 31006 Pamplona, Spain

**Keywords:** tomato (*Solanum lycopersicum* L.), heat stress, nitrate, ammonium, proline, photosynthesis, amino acids, polyamines, nitrogen metabolism

## Abstract

This study investigates how nitrogen form (100/0 NO_3_^−^ vs. 50/50 NO_3_^−^/NH_4_^+^), heat stress (43 °C), and foliar proline application interact to modulate the growth, photosynthesis, and nitrogen metabolism of two tomato cultivars (*Solanum lycopersicum* L.), ‘Cherry’ and ‘Tres Cantos’. Nitrogen-exclusive nutrition optimized biomass accumulation and photosynthetic efficiency. Conversely, mixed nutrition reduced photosynthetic performance but enhanced nitrogen storage and detoxification pathways, as evidenced by a shift in amino acid profiles (decreased glutamate and aspartate alongside increased glutamine and asparagine). Under heat stress, growth declined; however, plants exhibited non-stomatal photosynthetic acclimation. While exogenous proline failed to increase biomass, it significantly enhanced heat tolerance by driving transpiration and evaporative cooling. Cultivar-specific assessments revealed high metabolic plasticity in ‘Cherry’, whereas ‘Tres Cantos’ adopted a conservative strategy centered on the accumulation of protective nitrogenous compounds.

## 1. Introduction

Tomato (*Solanum lycopersicum* L.) is a crop of great economic, social, and nutritional importance, and is widely adapted to diverse climatic conditions and soil types. This versatility has enabled its integration into various agricultural systems, promoting production diversification and establishing it as a staple food in the Mediterranean diet, particularly in Spain. Consequently, the development of more efficient cultivation techniques has become a priority to increase productivity and reduce environmental impact [1].

In the current context of climate change, tomato cultivation faces significant challenges due to extreme environmental conditions. Rising temperatures and prolonged heat waves negatively affect plant physiological development, especially in arid and semi-arid regions. Heat stress, whose impact depends on the intensity, duration, and fluctuation of temperatures above the optimal range, causes several biochemical alterations, including protein denaturation and inhibition, protein degradation, and changes in the fluidity and integrity of lipid membranes [2]. In tomato plants, diurnal temperatures exceeding 35 °C and nocturnal temperatures above 21 °C compromise both production quantity and quality, accelerating fruit maturation while reducing optimal fruit size [3]. Physiologically, heat stress disrupts chloroplast photosynthetic machinery in tomato plants. It damages the photosystem II (PSII) complex, forcing excess electrons in the electron transport chain (ETC) to generate reactive oxygen species (ROS) [4,5]. Furthermore, heat-induced increases in leaf transpiration to aid thermoregulation can cause severe tissue dehydration without adequate irrigation, subsequently impairing mineral translocation and water use efficiency (WUE) [6,7]. Despite these general trends, heat stress tolerance and response mechanisms vary significantly among different tomato genotypes [3]. In particular, Cherry tomato genotypes are widely cultivated due to their high agronomic and commercial value, and are characterized by their small fruits, vigorous growth, and high market demand. Previous studies have shown that cherry tomatoes exhibit considerable physiological plasticity under abiotic stress conditions, including adjustments in growth, photosynthesis, and nutrient use efficiency [8,9]. Furthermore, Tres Cantos is a local cultivar traditionally grown under Mediterranean environmental conditions and represents a valuable genetic resource for evaluating adaptive responses to environmental constraints. The contrasting agronomic characteristics and genetic backgrounds of the Cherry tomato and “Tres Cantos” make them suitable models for investigating genotype-dependent physiological and metabolic responses to heat stress and nitrogen nutrition [10,11].

Various strategies have been proposed to mitigate the effects of several abiotic or biotic stresses, including heat stress. One such strategy involves the use of different nitrogen (N) sources [12,13,14]. Plants typically absorb N in two chemical forms: nitrate (NO_3_^−^) and/or ammonium (NH_4_^+^). Depending on the species, the effect of combined N nutrition on the tolerance to abiotic or biotic stresses may vary. For instance, some studies have reported that plants grown with NO_3_^−^ showed resistance to drought stress or pathogen attack [15] compared to NH_4_^+^-fed plants [16,17]. In contrast, several other works have reported increased resistance to drought or salt [13,18] or biotic stress [19] when plants were grown with higher concentrations of NH_4_^+^. Under heat stress, the application of NH_4_^+^-N in an appropriate nitrate-to-ammonium ratio has been shown to positively alleviate stress symptoms in crops such as cauliflower and baby-leaf lettuce [14,20].

Recent advances have highlighted the central role of transcription factors in bridging abiotic and biotic stress responses [21], and epigenetic mechanisms have been specifically implicated in tomato heat adaptation [22]. Previous studies have shown that nitrogen nutrition affects tomato responses to heat stress [23]. However, how nitrogen form interacts with genotype-specific metabolic responses and exogenous proline application to shape heat stress tolerance remains poorly understood. Addressing this knowledge gap requires a critical evaluation of the N source, balancing the universal preference for nitrate (NO_3_^−^) against the potential of ammonium (NH_4_^+^) to induce physiological toxicity [24,25]. Interestingly, while tomatoes are broadly classified as sensitive to NH_4_^+^, it has been shown that this susceptibility is not uniform [25]. Instead, NH_4_^+^ tolerance varies significantly among different tomato varieties [25], emphasizing the potential of tailoring nitrogen formulations to specific genotypes to optimize heat stress mitigation.

Beyond N source management, exogenous proline application significantly improves plant tolerance to various abiotic stresses, offering a promising tool to mitigate climate change effects on sensitive crops such as tomato [26,27]. Proline is a primary N metabolite that plays a key role in osmoregulation under stress [28]. Consequently, it has been associated with enhanced leaf water retention, improved photosynthetic performance, activated antioxidant defenses, and pH homeostasis [26,29].

In this context, this study hypothesizes that a balanced NO_3_^−^/NH_4_^+^ ratio, combined with exogenous foliar proline application, will synergistically enhance heat tolerance in tomato plants by improving physiological responses under heat stress conditions. For this purpose, we aimed to assess the response of primary N metabolism and the physiology of two tomato plant varieties (Cherry and Tres Cantos var.) to different NO_3_^−^/NH_4_^+^ ratios and foliar proline application under heat stress conditions. Specifically, variables related to growth, gas exchange, photosynthetic activity, and biochemical markers related to stress, such as amino acid content and polyamines, were analyzed. In addition, gas exchange behavior was evaluated using response curves to light intensity and CO_2_ concentration. More specifically, the following questions were asked: (i) how do these varieties differ in photochemical stability, carbon gain, and recovery at extreme temperature? (ii) how does a 50/50 NO_3_^−^/NH_4_^+^ ratio modulates stomatal conductance, A–C_i_ parameters, and photoinhibition relative to 100% NO_3_^−^ under that stress? (iii) to what extent does exogenous proline at 43 °C preserve Fv/Fm, sustain A–PPFD saturation, and improve intrinsic WUE? (iv) how do amino acids and polyamines track with photosynthetic impairment/maintenance and fluorescence signatures under heat stress through metabolic reprogramming? The last objective: (v) to determine whether heatmaps and principal component analysis (PCA) can robustly resolve the factorial treatments (genotype × temperature × nitrogen nutrition × exogenous proline) and uncover integrated trait complexes that predict thermotolerance.

## 2. Results and Discussion

### 2.1. Plant Growth

**Figure 1:**
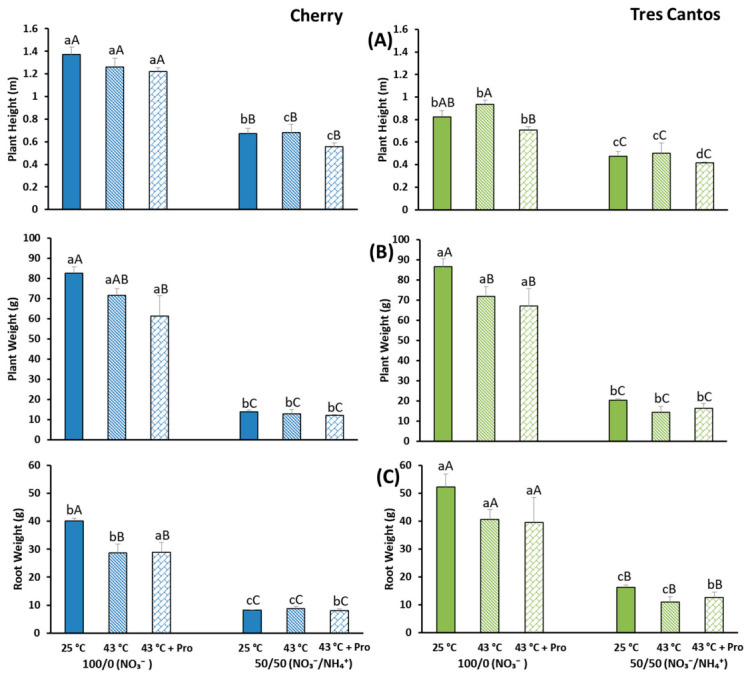
Effects of the nitrogen source 100/0 (NO_3_^−^) and 50/50 (NO_3_^−^/NH_4_^+^), temperature (25 °C and 43 °C), and foliar application of proline on plant height (**A**), plant weight (**B**), and root weight (**C**) in different varieties (Cherry and Tres Cantos) of tomato plants. Data are presented as Duncan test, mean ± SE (*n* = 5). Lowercase letters indicate the differences between the tomato varieties, and different uppercase letters indicate the differences between the various temperatures and the foliar application of proline within the same variety (*p* ≤ 0.05).

The nitrogen source, temperature, and foliar application of proline influenced the growth parameters of tomato plants, as shown in Figure 1. Nitrogen nutrition had a marked effect on the growth of the varieties. The all-nitrate 100/0 (NO_3_^−^) nutrient solution resulted in the highest values in plant height, weight, and root weight, while the combined 50/50 (NO_3_^−^/NH_4_^+^) nutrient solution significantly reduced all the evaluated parameters. These results indicate that at this concentration in the combined nutrient solution, the addition of NH_4_^+^ had a negative effect on plant development due to ammonium induced growth suppression processes, limiting vegetative growth and root development [24]. In Figure 1A, the Cherry variety showed a 66.5% increase in plant height compared to the Tres Cantos variety at 25 °C, with significant differences. However, in Figure 1B, corresponding to plant weight, the Tres Cantos variety remained slightly above Cherry in all treatments, behaving similarly in Figure 1C, which refers to root weight, although in the latter, the difference between varieties was more pronounced [30].

Heat stress (43 °C) generally decreased growth parameters compared to the 25 °C control, with a downward trend observed in plant weight (Figure 1B) and root weight (Figure 1C), consistent with heat-induced transmembrane damage and enhanced respiration rates [23]. Remarkably, the plant height of Tres Cantos remained statistically unaffected by the temperature increase, suggesting a lower vertical growth sensitivity to acute heat in this cultivar. While the foliar application of proline (43 °C + Pro and 100/0 NO_3_^−^) nutrition did not promote increases in shoot height or weight, it induced a crucial response in root biomass allocation. In Figure 1C, the addition of proline under heat stress shifted the cultivar significance, rendering its root weight statistically indistinguishable from that of Tres Cantos. According to the literature, this implies that exogenous proline may have preferentially protected the root system of the otherwise more heat-susceptible Cherry variety [23,31]. These results suggest that, under our experimental conditions, the foliar application of proline may have acted primarily on cellular protection and maintenance mechanisms against stress, rather than focusing on plant growth [32]. This response supports the role of proline in stabilizing proteins, maintaining redox homeostasis, and scavenging reactive oxygen species (ROS) in specialized sinks under severe abiotic stress, prioritizing cellular survival over expansive growth [33].

### 2.2. Gas Exchange Parameters

#### 2.2.1. Gas Exchanges Parameters to Different Light Intensities

**Figure 2:**
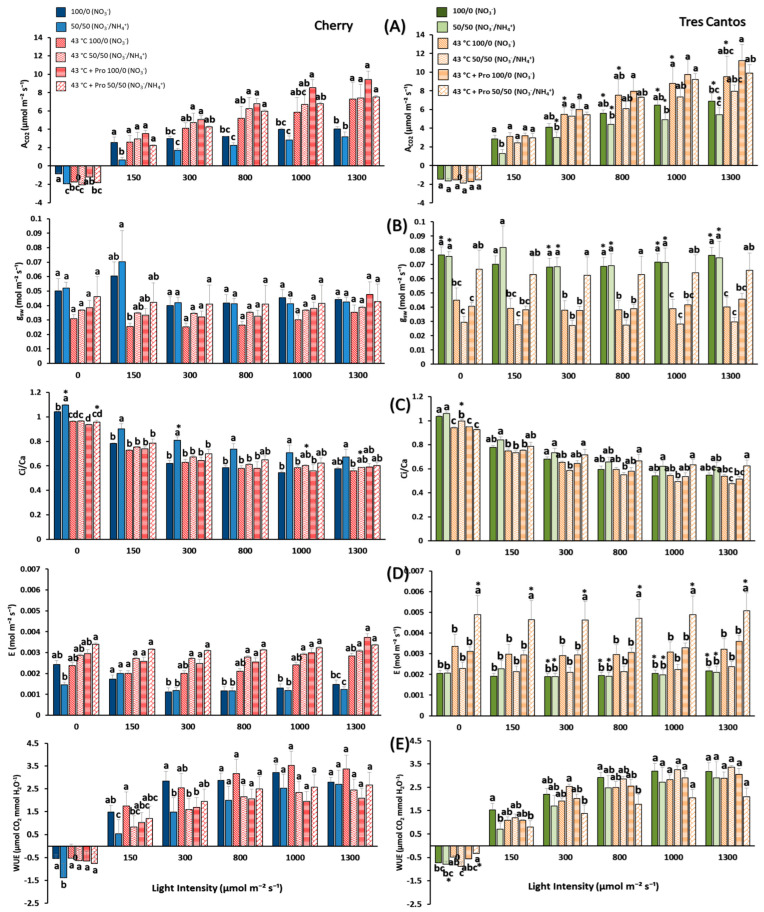
Response of gas parameters (A_CO_2__ (**A**), g_sw_ (**B**), Ci/Ca (**C**), E (**D**) and WUE (**E**)) to different light intensities in tomato plants under different nitrogen sources (NO_3_^−^ and NO_3_^−^/NH_4_^+^), heat stress conditions (43 °C) and exogenous application of proline. Lowercase letters mean significant differences between treatments within the same variety, and the (*) mean significant differences between varieties within the same treatment (*p* ≤ 0.05, Duncan test, mean ± SE (*n* = 5)).

Figure 2A shows how photosynthesis (A_CO_2__) increased progressively with light intensity, showing a trend toward higher values starting at 800 μmol m^−2^ s^−1^. Under control conditions, both varieties showed moderate A_CO_2__ values, although differences between varieties were observed starting at 300 μmol m^−2^ s^−1^, with Tres Cantos showing the most significant increases (*) over Cherry, at 38.09, 74.73, 61.94, and 71.11%, respectively. The 50/50 (NO_3_^−^/NH_4_^+^) combination reduced the values considerably within the same variety, but not between varieties, with Tres Cantos consistently demonstrating superiority. Heat stress increased A_CO_2__ values in both genotypes, suggesting some degree photosynthetic acclimatization to high temperatures.

Significant differences were observed in the Tres Cantos variety as compared to Cherry starting at 300 μmol m^−2^ s^−1^, when the nutrient solution contained only NO_3_^−^. The foliar application of proline under heat stress enhanced photosynthesis, reaching its maximum values in Tres Cantos at 1300 μmol m^−2^ s^−1^, with the Tres Cantos variety again showing higher values as compared to Cherry, at the highest light intensities.

The behavior of stomatal conductance (g_sw_) is shown in Figure 2B. The Cherry variety showed low to moderate values, with a slight decrease in the treatments with the combined N nutrient solution (NO_3_^−^/NH_4_^+^). The Tres Cantos variety showed a clearly higher g_sw_ (with significant differences between varieties) under control conditions, and a decrease in values was observed when the temperature increased to 43 °C, demonstrating heat-induced stomatal closure to minimize water loss. Proline foliation showed a tendency towards partial recovery of g_sw_ values, especially under high intensity conditions for both varieties.

The ratio between intercellular and ambient CO_2_ concentration (Ci/Ca) (Figure 2C) decreased progressively with increasing light intensity, indicating an increased CO_2_ demand possibly associated with greater photosynthetic activity and efficient RuBP (Ribulose-1,5-bisphosphate) carboxylation. Under control conditions (25 °C), the Cherry variety showed significant differences between plants irrigated with the combined N solution and those irrigated with a purely nitrate-based solution, unlike the Tres Cantos variety, for which no such differences were observed. The increase in temperature (43 °C) moderately accentuated the decrease, with differences between varieties observed at maximum light intensities (1000–1300 μmol m^−2^ s^−1^). Furthermore, the foliar application of proline tended to stabilize the Ci/Ca values, without showing significant differences between treatments or varieties.

Transpiration (E) values (Figure 2D) under the control conditions were similar within the treatments; however, the Tres Cantos variety showed higher values, with differences compared to the Cherry variety starting at 300 μmol m^−2^ s^−1^. With increasing temperature, E values increased progressively and significantly, driven by the drastic increase in leaf-to-air vapor pressure deficit (VPD), without showing significant differences, except for the plants treated with 50/50 (NO_3_^−^/NH_4_^+^) and proline foliation, which maintained significant differences within the treatment and between varieties.

Figure 2E shows an increasing trend in water use efficiency (WUE) values for both varieties as light intensity increased from 0 to 1000 μmol m^−2^ s^−1^. No significant differences were observed between treatments or between varieties. However, Cherry showed a greater variability in the results (although not statistically significant) compared to Tres Cantos, which exhibited a more consistent response. The highest values at low irradiance were observed in the control plants, and as the intensity increased, the highest values were obtained in the treatments subjected to heat stress.

The increase in carbon dioxide assimilation (A_CO_2__) at high temperature (43 °C) and high light intensities indicates that the photosynthetic apparatus remained functional and the plants experienced photosynthetic acclimatization rather than inhibition. Previous studies have shown that this combination could reflect the maintenance of photosynthetic biochemical capacity, including increased enzyme activity and efficient regeneration of photosynthetic intermediates, promoting adaptive responses and, consequently, improvements in photosynthetic performance in tomato plants [34]. This behavior can also be explained by the possible increase in carboxylation capacity, which is associated with a greater activation of the Rubisco (Ribulose-1,5-bisphosphate carboxylase/oxygenase) enzyme, through Rubisco activase [35]. Relating the data obtained from Ci/Ca, at 43 °C, indicates that CO_2_ fixation remained efficient, suggesting a balance between carboxylation and photorespiration [36].

Under a 50/50 (NO_3_^−^/NH_4_^+^) combined nutrition regime, the decrease in A_CO_2__ suggests that NH_4_^+^ interferes with photosynthetic efficiency. NH_4_^+^ absorption is accompanied by proton release, which acidifies the environment and, in turn, interferes with photosynthetic enzymes, diverting carbon towards amino acid synthesis. Consistent with previous studies, NH_4_^+^ reduced photosynthetic efficiency, while NO_3_^−^ promoted a better integration between carbon and nitrogen metabolism [37].

The increase in A_CO_2__ in plants treated with foliar proline at 43 °C reinforces the idea of the benefits of this method as a marker of tolerance to stress, protection of membranes and components of the photosynthetic apparatus, while proline also eliminates reactive oxygen species (ROS), thereby reducing the oxidative damage caused by the high temperature, allowing an increase in photosynthetic efficiency [38]. The literature highlights the importance of using biostimulants (amino acids in our case) to improve plants in the face of increasing environmental impact, due to their stimulation of defense mechanisms against stress [33].

The g_sw_ values obtained remained within a low range (≤0.01 mol m^−2^ s^−1^ under the light curve and ≤0.45 mol m^−2^ s^−1^ under CO_2_ curve); however, the decrease in Ci/Ca and the increase in A_CO_2__ as light intensity increased indicate that photosynthesis was not limited by stomatal constraints. On the contrary, the results point to a predominance of biochemical regulation, where the CO_2_ demand exceeded its diffusion rate, reflecting the aforementioned greater carboxylation efficiency rather than a limitation [39,40].

The increasing values of E under high light intensities and 43 °C indicate a greater evaporative demand and a cooling mechanism to prevent heat damage [41], although the low stomatal conductance (g_sw_) values due to stress suggest that water loss was partially modulated by non-stomatal cuticular transpiration or hyper-evaporation driven by the extreme heat gradient. High temperatures intensify the evaporation gradient, promoting water loss even when g_sw_ is restricted [42]. Proline application at high temperatures increased E values, suggesting an enhanced capacity to sustain water fluxes and physiological functioning under heat stress conditions [27].

The WUE values at 43 °C indicate that carbon assimilation managed to balance the water loss caused by the temperature increase, thus implying a relative equilibrium between carbon assimilation and water loss fluxes [43]. On the other hand, in plants treated with proline, WUE values were lower at some high intensities, suggesting a change in this relationship. Considering the benefits of proline, it is reasonable to assume that with higher energy expenditure E values, aimed at maintaining cellular hydration and metabolic activity, the ratio between carbon dioxide (A_CO_2__) and water loss decreases. This demonstrates that WUE values represent a balance between carbon assimilation and water loss, and that they are, in turn, are influenced by stress and applied protective compounds [44].

#### 2.2.2. Gas Exchanges Parameters to Different CO_2_ Concentrations

**Figure 3:**
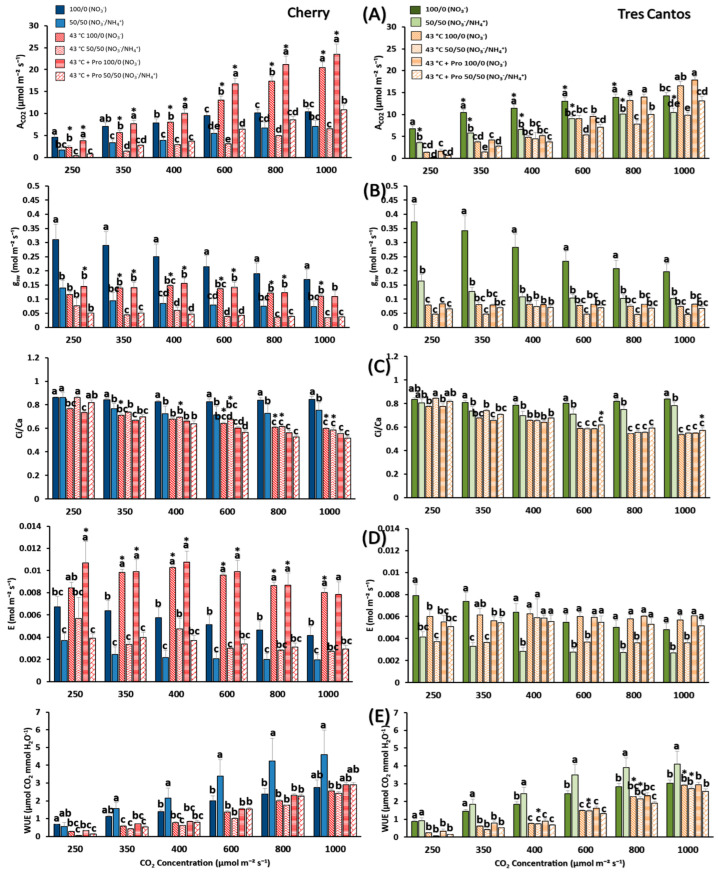
Response of gas parameters (A_CO_2__ (**A**), g_sw_ (**B**), Ci/Ca (**C**), E (**D**) and WUE (**E**)) to different CO_2_ concentration in tomato plants under different nitrogen source (NO_3_^−^ and NO_3_^−^/NH_4_^+^), thermal stress conditions (43 °C) and exogenous application of proline. Lowercase letters mean significant differences between treatments within the same variety, and the (*) mean significant differences between varieties within the same treatment (*p* ≤ 0.05, Duncan test, mean ± SE (*n* = 5)).

The increase in CO_2_ concentration in turn increased A_CO_2__ (Figure 3A) in both varieties. In contrast to the light curve, the Cherry tomato values were significantly higher than those observed in the Tres Cantos variety under heat stress conditions, reaching maximum assimilation rates near 25 μmol m^−2^ s^−1^. Under 100/0 (NO_3_^−^) nutritional conditions, a progressive increase was observed, from 250 to 1000 µmol m^−2^ s^−1^ of CO_2_. In the Cherry variety, heat stress (43 °C) significantly increased A_CO_2__ values starting at 600 µmol m^−2^ s^−1^. This did not occur in the Tres Cantos variety, with the same trend as the control condition observed as the CO_2_ concentration increased. The application of proline caused a further increase, reaching the highest A_CO_2__ values, especially in the Cherry variety. On the other hand, the combined 50/50 (NO_3_^−^/NH_4_^+^) nutrition showed significantly lower values in relation to the 100% NO_3_^−^ nutrition across all CO_2_ levels, highlighting that CO_2_ enrichment cannot fully rescue the photosynthetic apparatus from ammonium toxicity.

The g_sw_ (Figure 3B) showed a pronounced decrease in its values with increasing CO_2_ concentration. The Tres Cantos variety, under the control conditions, presented higher values (without significant differences) as compared to the Cherry variety. Increased temperature reduced g_sw_ values, especially in the 50/50 (NO_3_^−^/NH_4_^+^) combined nutrient regime and in the Tres Cantos variety. Proline foliation did not change the effect of heat stress, but only tended to keep values slightly higher.

At 25 °C, the ratio between internal and external CO_2_ concentration (Ci/Ca) showed a moderate tendency to stabilize as ambient CO_2_ increased (Figure 3C), maintaining a similar behavior in both varieties. Furthermore, it is worth noting that, starting at a CO_2_ concentration of 350 µ mol m^−2^ s^−1^, the difference between the two nutrient regimes became more pronounced, with a decrease observed in the combined regime compared to the nitrate-only regime. Heat stress caused further reductions in the Ci/Ca values, behaving similarly in both varieties.

The transpiration rate (E) (Figure 3D) at 25 °C in both varieties decreased, with significant differences between nutrient levels. The 100/0 (NO_3_^−^) treatment obtained higher values at all CO_2_ concentrations tested. Heat stress significantly increased the values in the Cherry variety starting at a CO_2_ concentration of 250 µmol m^−2^ s^−1^, where E doubled compared to its respective control. In the case of the Tres Cantos variety, the values obtained at 43 °C were higher than the control condition starting at 600 µmol m^−2^ s^−1^, with no significant differences observed between treatments, except for the combined nutrient solution.

In both varieties, water use efficiency (WUE) (Figure 3E) increased along with CO_2_ concentration. However, the 50/50 (NO_3_^−^/NH_4_^+^) combined nutrient solution at 25 °C showed higher values compared to the 100/0 (NO_3_^−^) nutrient solution. The effect of heat stress caused a decrease in values compared to the control (although they continued to increase as CO_2_ concentration increased). Proline application showed a slight increase compared to the treatments without foliar application.

The increase in temperature led to a continuous increase in A_CO_2__ in both varieties under high CO_2_ concentration. These values suggest that photosynthetic carbon assimilation remained functional under our experimental conditions and may reflect a degree of photosynthetic acclimation to heat stress, consistent with previous studies conducted on C3 plants [45]. Another important point is the high availability of CO_2_, which allowed A_CO_2__ to continue increasing despite the temperature, helping to promote carbon fixation and the reduction of photorespiration [46].

The observed reduction in g_sw_ values and the increase in temperature show how, in response to this stress (43 °C), the stomata tend to limit water loss under conditions of increased evaporative demand, which allows the plant to maintain its water balance [47]. The conditions of increased CO_2_ concentration somewhat compensated for the limitations caused by the partial closure of stomata, due to their ability to fix carbon even when gas intake is moderately restricted [48].

The increase in CO_2_ concentration at 25 °C resulted in an efficient regulation of the Ci/Ca ratio, reflecting a greater internal availability of CO_2_ that matched the accelerated carboxylation [46]. In contrast, when the temperature increased to 43 °C, the Ci/Ca ratio decreased, along with g_sw_, although this was accompanied by an increase in A_CO_2__. This behavior may indicate that under stress, stomatal closure, and reduced diffusive flux, the progressive increase in CO_2_ compensated for this restriction, causing a decrease but not a photosynthetic limitation [49].

Transpiration (E) was observed to have different values among treatments and varieties. The increase in temperature increased evaporative demand, although different genotypes may respond differently to this increase [50]. Cherry tomato, on its part, used a strategy based on evaporative cooling (showing higher values), allowing it to decrease leaf temperature by dissipating latent heat at high temperatures. This behavior has been associated in previous studies with heat-tolerant genotypes [50]. On the other hand, Tres Cantos showed a more moderate response, limiting water loss. This strategy is more consistent with heat-sensitive genotypes, which use water conservation as their primary strategy [51,52].

Interestingly, under control conditions (25 °C) and 50/50 (NO_3_^−^/NH_4_^+^) combined nutrition, water use efficiency (WUE) showed the highest values, exceeding that of the 100/0 (NO_3_^−^) nutrition regime. This behavior may be due to the reduction in stomatal conductance (g_sw_) caused by NH_4_^+^, which limits transpiration, leading to an increase in the A_CO_2__/E ratio [53]. On the other hand, under heat stress (43 °C) a decrease in WUE was observed in both varieties. This can be explained by the fact that despite the increase in A_CO_2__ with temperature and CO_2_ concentration, the increase in evaporative demand was greater, causing this relationship to lead to a reduction in water efficiency [54].

### 2.3. Amino Acid and Polyamine Profile Content

#### 2.3.1. Amino Acid Profile

A metabolomic analysis allowed the detection and quantification of 28 amino acids (AA) (Table 1) in tomato plant leaves (*Solanum lycopersicum*). These AA are involved in both protein synthesis and in the physiological response to stress. They are classified as proteinogenic (the predominant ones) and non-proteinogenic. Proteinogenic amino acids are part of cellular proteins and are essential for plant growth and development, as they are part of structural, enzymatic, and transport proteins. They are also linked to the available Nitrogen (N) source, since it is a fundamental component of their structure. Non-proteinogenic amino acids are not incorporated into proteins; they function as metabolic intermediates, signaling molecules, or nitrogen reserve compounds and are precursors for the biosynthesis of primary and secondary metabolites, which are essential for defense against stress (Table 1) [55,56].

The AA profile was dominated by glutamic acid (Glu), glutamine (Gln), and aspartic acid (Asp), which together represented approximately 44–48% of the total AA content. This predominance of Glu and Gln with temperature changes reflects their central role in nitrogen metabolism, as their concentration is associated with the assimilation and recycling of NH_4_^+^. In turn, Asp increases due to the donation of amino groups provided by glutamate [57].

At the individual level, the combined N supply (NO_3_^−^/NH_4_^+^) induced a reduction in Glu content in both tomato genotypes, with the extent depending on the temperature and the treatment applied. Under control conditions (25 °C), Glu decreased slightly in Cherry tomato from 7.19 to 7.04 µmol/gDW (2.13%), but more markedly in Tres Cantos, from 8.58 to 6.18 µmol/gDW (38.83%) relative to the control treatment value. However, these variations at 25 °C indicate that the basal pool of glutamate remained highly homeostatically regulated under optimal temperatures regardless of the nitrogen source. Heat stress (43 °C) significantly intensified the decrease in Glu in both cultivars (97.38% in Cherry and 81.93% in Tres Cantos), as compared to their respective 43 °C nitrate controls. Remarkably, heat stress alone under exclusive NO_3_^−^ nutrition triggered a major accumulation of Glu, peaking at 15.85 µmol/gDW in Cherry and 16.41 µmol/gDW in Tres Cantos, suggesting a heat-induced activation of transamination pathways. When proline was applied under heat stress (43 °C + Pro), Glu decreased even further in Cherry (112.59%), while the reduction was completely mitigated in Tres Cantos (22.49%). However, despite these numerical oscillations between individual means (simple ANOVA), the multi-factorial ANOVA (A × B) indicated that these shifts were not statistically significant within any of the three heat treatments. This could reveal that the fundamental glutamate backbone is highly buffered and that the plant maintains a strict structural homeostasis against independent variations in nitrogen forms within each environment.

Gln showed significant differences (Table 2) depending on the nitrogen source in both varieties, showing a clear stimulation under combined N supply (NO_3_^−^/NH_4_^+^) compared to exclusive NO_3_^−^ nutrition. At 25 °C, the Cherry variety showed a very high relative increase of 332.98% (8.52 to 36.89 µmol/gDW), while Tres Cantos showed an increase of 144.05% (from 11.26 to 27.48 µmol/gDW), indicating a higher initial capacity for NH_4_^+^ assimilation in the Cherry variety. Under heat stress (43 °C), the accumulation of Gln was even more pronounced (85.75 µmol/gDW in Cherry and 72.15 µmol/gDW in Tres Cantos). In the combined 43 °C + Pro treatment, although Gln values remained higher compared to exclusive NO_3_^−^ nutrition (150.25% in Cherry and 91.78% in Tres Cantos), a relative decrease was observed compared to the 43 °C treatment without proline.

The decrease in Glu pools relative to Gln under ammonium nutrition is consistent with increased ammonium assimilation possibly through the GS/GOGAT (Glutamine synthetase/glutamate synthase) cycle. Under NH_4_^+^ nutrition, glutamine synthetase (GS) incorporates ammonium into Glu to form glutamine (Gln), which is subsequently converted back into Glu by glutamate synthase (GOGAT). The increase in NH_4_^+^ availability likely stimulates GS activity, promoting Glu consumption and Gln accumulation. Therefore, the observed reduction in Glu is closely linked to the concomitant increase in Gln and reflects an active detoxification and re-assimilation mechanism [58].

Asparagine (Asn) concentrations rose steeply under the combined heat and ammonium stress (43 °C + 50/50) treatment, increasing from baselines below 2.16–4.63 µmol/gDW up to 18.26 µmol/gDW in Cherry and 21.71 µmol/gDW in Tres Cantos. Consistent with the literature, this indicates that the AS (Asparagine Synthetase) pathway acts as a secondary, highly efficient metabolic sink for nitrogen storage and transport under severe stress conditions when the capacity of the GS/GOGAT cycle faces biochemical pressure [58].

The marked decrease of Glu at 43 °C when that ammonium concentration was introduced suggests greater metabolic pressure to avoid ammonia toxicity and maintain nitrogen homeostasis [24]. The differential response to exogenous proline indicates different regulatory strategies. Overall, the results indicate that nutrition with NH_4_^+^, especially under heat stress, promotes a shift in nitrogen metabolism towards the accumulation of Gln and Asn at the expense of Glu [59]. This adjustment reflects a coordinated mechanism for ammonium detoxification and maintenance of nitrogen homeostasis. The differences between Cherry and Tres Cantos suggest contrasting metabolic strategies, with Cherry showing a greater plasticity in Gln accumulation, while Tres Cantos seems to maintain a more conservative balance under combined stress [60].

Proline (Pro) showed a significant increase under combined nutrition (50/50 NO_3_^−^/NH_4_^+^) in both varieties, although with different magnitudes depending on the temperature. This multi-level plasticity is fully supported by the multi-factorial ANOVA, which confirmed significant main effects for variety, nitrogen source, and a significant interaction across all temperature regimens. At 25 °C, the relative up-regulation compared to the nitrate control was more pronounced in Cherry (609.86% increase reaching 5.04 µmol/gDW) than in Tres Cantos (110.94% reaching 1.35 µmol/gDW), signaling a substantial baseline metabolic disruption in Cherry under ammonium nutrition. Under heat stress (43 °C), Pro accumulation remained high (121.65% in Cherry and 86.05% in Tres Cantos). In the (43 °C + Pro) treatment, the exogenous application naturally increased baseline values in the nitrate controls (6.03 and 7.79 µmol/gDW, respectively) and the relative increase in the combined (50/50) treatment was lower in Cherry (40.96%), while in Tres Cantos, a decrease was observed (20.40%). These results suggest a greater metabolic sensitivity of Cherry to the presence of NH_4_^+^ even in the absence of heat stress. This pattern aligns with previous studies that described proline accumulation not only as a response to abiotic stress, but also due to imbalances in nitrogen metabolism. Proline contributes to membrane protection, protein stabilization, and maintenance of redox balance, functions that are particularly relevant when heat stress increases the generation of reactive oxygen species (ROS) [61]. Overall, the results confirm that proline accumulates under stress conditions and that its concentration is enhanced by a higher proportion of NH_4_^+^ in the nutrient solution [60,62].

Furthermore, other stress-responsive amino acids followed this hyper-accumulation pattern; for instance, Lysine (Lys) levels increased dramatically under combined heat and ammonium nutrition (43 °C + 50/50), reaching 10.87 µmol/gDW in Cherry and 8.62 µmol/gDW in Tres Cantos, governed by significant impacts in variety and nitrogen source, and their interaction indicating a generalized activation of metabolic shunts or accelerated protein catabolism under concurrent stresses. This highlights a uniform, species-wide metabolic commitment toward channeling excess ammonium into organic nitrogen forms to preserve cellular survival at the expense of expansive vegetative growth.

Asp, just as Glu, showed variations under combined nutrition compared to the exclusive supply of NO_3_^−^. Specifically, Asp pools exhibited a strong increase under the (50/50) combined nitrogen regime at 25 °C, but experienced a decline under heat stress (43 °C). Crucially, the multi-factorial ANOVA revealed these fluctuations to be entirely non-significant across all factors (Variety A, Nitrogen source B, and their interaction). This strict homeostatic buffering of the aspartate substrate pool contrasts sharply with asparagine (Asn), which showed a highly significant increase exclusively driven by the nitrogen source independent of the variety. The increase in Gln provides the amide group necessary for the activation of asparagine synthetase (AS), the enzyme responsible for converting Asp into Asn [63]. Consequently, the strong accumulation of Asn is coupled with the metabolic flux utilizing Asp as a substrate, although downstream transamination continuously replenishes the free Asp pool to preserve its stability. Asn constitutes a key molecule for nitrogen storage and transport due to its high N/C (Nitrogen/Carbon) ratio and lower reactivity as compared to free NH_4_^+^. Therefore, its accumulation under ammonium nutrition represents an efficient metabolic strategy to avoid ammonium toxicity. These results are consistent with previous studies that described the coordinated accumulation of Gln and Asn as an adaptive mechanism in response to ammonium nutrition and stress conditions, reflecting a reorganization of amino acid metabolism aimed at detoxification and safe nitrogen storage [60].

Regarding the differences between varieties, in general terms, both showed a similar overall profile in AA composition, corroborated by the non-significant effect on primary pools such as Glu, Gln, Asp, Asn, and Total AA. Nevertheless, a highly significant variety effect was isolated for specific stress-responsive amino acids, including Proline, Lysine, Histidine, Citrulline, beta-aminoisobutyric acid, and Homocystine.

The contrasting amino acid profiles suggest distinct adaptive strategies in the two cultivars. Under heat stress, Tres Cantos accumulated higher levels of nitrogen-rich amino acids, particularly amide compounds such as glutamine and asparagine, indicating a greater capacity for nitrogen assimilation, storage, and remobilization to maintain C/N homeostasis under adverse conditions. In contrast, Cherry exhibited more pronounced changes in amino acid composition following proline application, suggesting a more flexible metabolic response involving the reallocation of carbon and nitrogen resources and a rapid adjustment of amino acid metabolism. These results indicate that Tres Cantos primarily relies on nitrogen conservation mechanisms, whereas Cherry appears to respond through enhanced metabolic plasticity under combined heat and nutritional stress [64,65].

**Figure 4:**
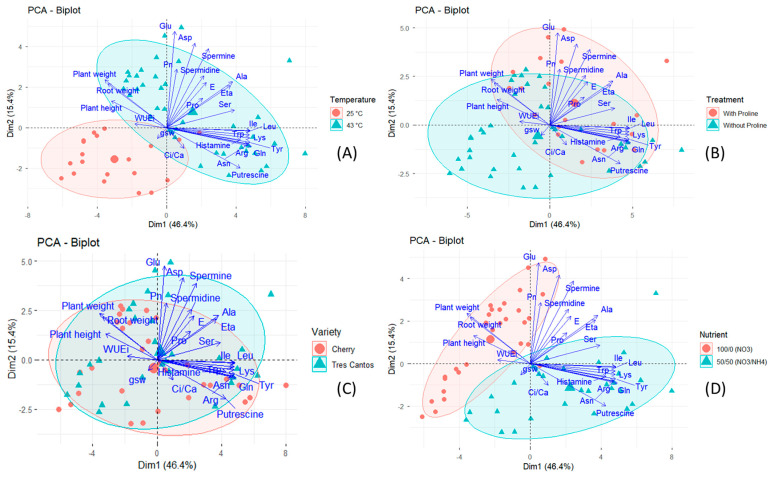
Principal component analysis (PCA) showing the effects of temperature (**A**), proline application (**B**), variety (**C**), and nitrogen source (**D**) on physiological traits and amino acid and polyamine profiles in tomato plants.

**Figure 5:**
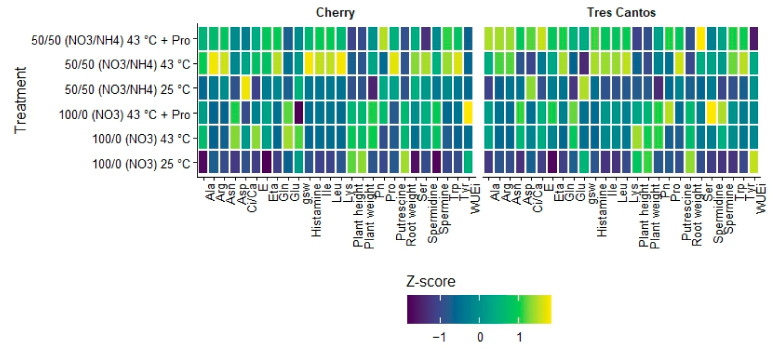
Heat map showing the effects of temperature, proline application, variety, and nitrogen source on physiological traits and amino acid and polyamines profiles in tomato plants.

The combined N nutrition (NO_3_^−^/NH_4_^+^) showed a high statistical significance in most cases (*p* ≤ 0.001) (Table 2). Supplementation with NH_4_^+^ promoted the accumulation of most AA compared to exclusive nitrate nutrition. A × B interactions were less frequent and generally of lower significance (*p* ≤ 0.05) [58]. Furthermore, increasing the temperature from 25 °C to 43 °C also resulted in a widespread and significant increase in the total free amino acid content (*p* ≤ 0.001). Figure 4 and Figure 5 further illustrate the association of these AA with temperature and their greater accumulation under stress conditions. These results suggest that the presence of NH_4_^+^ favors direct nitrogen assimilation and increased amino acid synthesis, especially under the applied conditions, given the high metabolic demand for protective nitrogen compounds [66].

The foliar application of proline at 43 °C had a marked effect on specific AA, although it did not substantially alter the overall AA profile. A significant increase was observed in both varieties, confirming the efficacy of the exogenous application and agreeing with previous studies that highlighted the relevance of amino acid foliar applications as a strategy to improve plant response to adverse conditions [27].

Interestingly, the impact of exogenous proline on the total amino acid content was treatment- and cultivar-dependent under the (50/50) combined nitrogen regime. Proline application induced a reduction in Total AA only in Cherry (decreasing from 216.38 to 179.65 µmol/gDW, whereas Tres Cantos remained highly stable 194.73 vs. 196.97 µmol/gDW. Conversely, under exclusive nitrate nutrition (100/0), the foliar treatment slightly increased the Total AA pool in both genotypes. This behavior suggests that the foliar application of proline does not induce a non-specific or massive accumulation of nitrogen compounds, but rather promotes a more efficient and targeted metabolic reorganization. Furthermore, exogenous proline may act as a regulator of the carbon-nitrogen distribution, influencing amino acid metabolism and the allocation of metabolic resources under thermal stress conditions. The selective accumulation of specific amino acids observed in the present study suggests a coordinated metabolic reprogramming, rather than a generalized increase in nitrogen-containing compounds [67,68]. Such responses may help maintain metabolic homeostasis and improve stress acclimation by redirecting carbon and nitrogen fluxes toward protective pathways, as proposed by [69] for stress-resistant crops.

In general, essential amino acids remained relatively unchanged in terms of global profile trends; this stability is related to their more conservative regulation, due to their mechanism for preserving cellular functions under unfavorable conditions [70]. In the case of non-essential amino acids, specifically those directly linked to the GS/GOGAT cycle, namely Gln, Glu, Asn, and Asp, these showed the greatest variations under the applied conditions, confirming their role in nitrogen assimilation and redistribution [59,71]. Proline exhibited a differential response to environmental stress as a non-essential amino acid. While it has always been considered a compatible osmolyte, previous studies have expanded its impact on processes related to stress, metabolic recovery, and the regulation of cellular signaling [72]. As for the derivatives, their accumulation may be associated with additional adjustments in alternative metabolic pathways to maintain cellular stability and carbon-nitrogen balance [73].

#### 2.3.2. Polyamine Profile

In tomato leaves, the chromatographic profile of polyamines (PA) (Table 3) consisted of histamine, putrescine, cadaverine, spermidine, and spermine. The profile was primarily dominated by putrescine, spermidine, and spermine, representing 63% to 97% in the different varieties and conditions tested.

Putrescine (Put) showed moderate increases under exclusive nitrate nutrition (2.27% in Cherry and 57.14% in Tres Cantos); while under combined nutrition, the increases were considerably greater (106.19% in Cherry and 162.38% in Tres Cantos). However, the temperature increase from 25 °C to 43 °C under exclusive NO_3_^−^ nutrition did not cause a statistically significant change in Put pools for either cultivar. Instead, a significant upward shift was restricted to the combined (50/50) nutrition regime in the Cherry variety.

Histamine (His) showed a similar behavior, with notable increases in both Cherry (225.0% and 350.0%) and Tres Cantos (162.5% and 379.31%). Regarding the higher molecular weight polyamines, spermidine (Spd) increased more moderately (45.31% and 32.22% in Cherry; 40.68% and 19.51% in Tres Cantos), while spermine (Spm) showed more variable increases (205.26% and 35.0% in Cherry; 196.0% and 109.68% in Tres Cantos). The accumulation of PA under 43 °C with combined nutrition (NO_3_^−^/NH_4_^+^) is consistent with previous studies that associated high polyamine concentrations with a greater capacity to tolerate adverse environmental conditions. PA participate in the stabilization of membranes and macromolecules, regulation of ionic balance, modulation of cell signaling, and protection against oxidative stress [74]. In particular, authors have noted an increase in the PA profile at high temperatures during the same time period in cauliflower cv. Moonshine [75].

The analysis of factor A (variety) revealed significant differences in specific PA under heat stress conditions (Table 4). The Tres Cantos variety exhibited higher levels of putrescine (Put) under exclusive nitrate nutrition (100/0 NO_3_^−^) at 43 °C, reaching values 22.22% higher than those observed in Cherry. Similarly, in the case of spermine (Spm), Tres Cantos showed a 27.59% increase compared to Cherry under the same heat conditions. Cherry, on the other hand, showed differences in putrescine (*p* ≤ 0.001) under combined nutrition (50/50 NO_3_^−^/NH_4_^+^) at 43 °C, where a more marked accumulation of this PA was observed compared to Tres Cantos. The varietal differences observed in the polyamine profile suggest contrasting adaptive strategies in response to heat stress. This behavior indicates an intrinsic ability of the genotype to mobilize cellular protection pathways against high temperatures [74]. The predominant increase in putrescine, accompanied by more moderate increases in spermidine (Spd) and spermine (Spm), suggests the activation of the enzyme arginine decarboxylase (ADC), responsible for the conversion of arginine to putrescine. The activation of this pathway is characteristic of plants subjected to heat stress and constitutes a rapid response mechanism to adverse conditions.

The foliar application of proline at 43 °C modified the PA profile; the total content was similar to or lower than that observed under heat stress without application, particularly under the (50/50 NO_3_^−^/NH_4_^+^) combined nutrition. At the individual level, a reduction in Put was recorded, while Spd and Spm remained the same or increased slightly, especially in the Tres Cantos variety. These changes did not imply a higher overall accumulation, but rather a redistribution in the relative proportion of the different PA [75].

### 2.4. Principal Component Analysis (PCA) and Heatmap Analysis of Physiological and Metabolic Responses in Tomato Plants Under Different Environmental and Nutritional Conditions

The principal component analysis (PCA) (Figure 4) shows the distribution of the samples and the contribution of the variables to the applied factors, namely: temperature (25 and 43 °C); foliar treatment (with or without proline); variety (Cherry and Tres Cantos); and nutrients (100/0 NO_3_^−^ and 50/50 NO_3_^−^/NH_4_^+^). The total variation was 61.8%, distributed as PC1: 46.4% and PC2: 15.4%, indicating that the first two components capture more relevant information, allowing for adequate discrimination. This analysis clearly shows that the main factor determining the separation between the samples was temperature (43 °C), associated with a greater accumulation of amino acids (AA) and polyamines (PA). Nutrition also exerted a significant difference: the 100/0 (NO_3_^−^) solution tended towards growth parameters, while the 50/50 (NO_3_^−^/NH_4_^+^) solution tended towards the accumulation of AA and PA. The application of proline exerted secondary modulating effects, and the varietal differences indicated differences in the plants’ response to stress.

The heat map (Figure 5) presents the values normalized using the Z-score. Warmer colors indicate a greater accumulation of the variables, while cooler colors indicate lower accumulation values. This analysis also shows that heat stress induced a metabolic reprogramming toward the accumulation of nitrogen compounds.

## 3. Materials and Methods

### 3.1. Experimental Design and Treatments

Tomato plants (*Solanum lycopersicum*) var. Cerasiforme (Cherry) and Solanaceae (Tres Cantos) (Batlle Huerto y Jardín, Barcelona, Spain) were germinated in commercial seed trays (El Jimenado S.A., Torre-Pacheco, Murcia, Spain). Tres Cantos tomato is a traditional Spanish cultivar, characterized by a tall vine and an indeterminate growth habit, with high productivity. The fruits of Tres Cantos are rounded and can weigh up to a quarter of a kilogram per fruit. By contrast, a commercial Cherry-type tomato is small (typically 8–25 g per fruit) and borne in compact clusters. Thus, Tres Cantos is described as highly productive with large individual fruits while Cherry-type cultivars generally achieve high fruit counts per plant with smaller individual fruits, enabling continuous harvest of multiple trusses but a lower mass per fruit.

After three weeks germination, they were transplanted into 5-L pots filled with perlite (74% total silicon, particle size 3–6 mm, and density 104 g/L) (Global Perlita, Almería, Spain) as a substrate. For irrigation, two modified Hoagland nutrient solutions were used. The control treatment contained (in mM): NO_3_^−^: 8.20; K^+^: 5.50; Ca^2+^: 2.10; SO_4_^2−^: 1.25; Mg^2+^: 0.5; P: 1.47. The other nitrogen source used was in the form of 50% NO_3_^−^/ 50% NH_4_^+^, where ammonium was introduced as (NH_4_)_2_SO_4_ with the formulation: NO_3_^−^: 4.20; NH_4_^+^: 4.0; K^+^: 5.50; Ca^2+^: 2.10; SO_4_^2−^: 5.25; Mg^2+^: 0.5; P: 1.47. The pH irrigation solutions were maintained in a range of 5.6–5.8 and with a 30–40% drainage percentage.

The cultivation was carried out in a climate chamber as described by [76], under controlled environmental conditions: photoperiod of day/night (16/8 h), temperatures of 25/20/18 °C (12/4/8 h), relative humidity of 60–70%, and photosynthetically active radiation (PAR) of 250 μmol m^−2^ s^−1^ [77]. Plants were maintained under these conditions for five weeks.

After this time of growth under control conditions (eight weeks after germination) and at the vegetative pre-flowering stage, plants were subjected to the different treatments. Proline was applied once, to one-third of the plants of each variety by spraying the leaves until runoff, ensuring uniform coverage of the leaves (approximately 45 mL per plant). The solution was prepared beforehand at a concentration of 2.5 mM, this concentration was selected based on previous studies reporting beneficial effects of exogenous proline on plant tolerance to abiotic stress [26,27], while the remaining plants received only distilled water. The plants were subjected to heat stress for three days with temperatures of 43/32/25 °C, maintaining the main photoperiod.

A completely randomized experimental design was used, considering the cultivar (Cherry and Tres Cantos), irrigation solution (100/0 NO_3_^−^ and 50/50 NO_3_^−^/NH_4_^+^), and treatments (control, heat stress and heat tress with proline application). Each plant grown individually in a pot was considered an independent biological replicate. Five biological replicates were included for each treatment combination, resulting in 60 experimental units (2 cultivars × 6 treatment combinations × 5 replicates). For biochemical determinations, samples obtained from each biological replicate were processed and analyzed separately.

### 3.2. Plant Growth

The height of the tomato plants was measured with a measuring tape, recording the distance from the base of the stem to the highest leaf, excluding the root system. At the end of the experiment, the plants were harvested and separated into different organs. The fresh weight of the shoot (stem and leaves), excluding the root, as well as the fresh weight of the root system, was determined. After the measurements, the plant samples were separated by organ and stored (at −80 °C for fresh matter and for dry matter, freeze-dried using an Alpha 1-4 LDplus freeze-dryer (CHRIST, Osterode am Harz, Germany) until further analysis.

### 3.3. Gas Exchange Parameters

A LI-6800 Portable Photosynthesis system (LI-COR, Lincoln, Nebraska, USA, and Software Version 2.0) was used to measure Gas exchange parameters. The A/I curve (carbon assimilation as a function of light intensity) gas exchange measurements were performed on one fully expanded young leaves per biological replicate, that had been pre-adapted to darkness for 10–20 min, following the instructions with the modifications proposed by [78]. Subsequently, the leaves were exposed to a sequence of increasing light intensities, ranging from 0 to 1300 μmol m^−2^ s^−1^ (0, 300, 600, 800, 1000 and 1300), while maintaining a constant CO_2_ concentration of 400 μmol mol^−1^. Measurements were recorded over a period of 60–120 s to allow the photosynthetic rate (A_CO_2__) to reach a steady state.

For the A_CO_2__/C curve (carbon assimilation as a function of CO_2_ concentration), leaves were subjected to a sequence of increasing CO_2_ concentrations, ranging from 250 to 1000 μmol mol^−1^ (250, 350, 400, 600, 800, and 1000), while maintaining a constant light intensity of 1300 μmol m^−2^ s^−1^. As in the previous measurements, data were recorded over 60–120 s to ensure A_CO_2__ reached a steady state.

In addition, the values of stomatal conductance (gs), intercellular CO_2_ concentration (Ci), and ambient CO_2_ concentration (Ca), transpiration rate (E) and water use efficiency (WUE) were obtained.

### 3.4. Amino Acid and Polyamine Profile Analysis

The amino acid (AA) and polyamines (PA) profiles were determined following the method from [79] with modifications. The compounds were extracted from previously lyophilized and powdered leaves, by mixing the dry sample with HClO_4_ (0.6 M), then vortexing, inverting (30 min), sonicating, and centrifuging the samples at 10,000 rpm for 10 min at 4 °C to extract the first supernatant. Subsequently, the same procedure was repeated to extract the second supernatant. The first and second supernatants were mixed and derivatized. For the derivatization, 70 µL of AccQ-Tag TM (Borate buffer) was used in a 10 µL of sample. Then, 20 µL of the derivatizing agent AQC (6-aminoquinolyl-N-hydroxysuccinimidyl carbamate) was added. This mixture was vortexed and then heated to 55 °C for 10 min. After heating, a portion was used for injection into an ACQUITY UPLC (Waters, Milford, MA, USA) equipped with a QTOF (Xevo-G2XS-Qtof) and a CORECSTM Premier C18 column (1.6 µm 2.1x150 mm). The flow rate was 0.28 mL min^−1^, the temperature was maintained at 55 °C, and the injection volume was 2 µL. The solvents used were: (A1), water + 0.1% Formic Acid and (B1), Acetonitrile + 0.1% Formic Acid. The gradient conditions applied were: 0–0.54 min 99.9% A1; 2 min 98% A1; 6 min 98% A1; 13 min 92% A1; 16 min 90% A1; 20 min 78% A1; 21.50 min 50% A1; 22 min 99.9% A1 and 23 min 99.9% A1. The mass spectrometer used the positive electrospray ionization mode with a sweep range between 100 and 1200 m/z. For the quantification of the AA and PA profiles, internal standards (Thermo Scientific, Waters, Milford, MA, USA) were used.

### 3.5. Statistical Analysis

To evaluate the effect of the experimental treatments, the data obtained were analyzed statistically, starting with the verification of assumptions of homogeneity of variance before performing the comparative analyses. Next, an analysis of variance (ANOVA) was performed to determine the significant differences between treatments. Subsequently, the means were compared using Duncan’s multiple range test, considering a significance level of *p* ≤ 0.05. In addition, a multivariate analysis was performed using Principal Component Analysis (PCA) with the aim of reducing the dimensionality of the data set and exploring the relationships between the variables analyzed. Heat maps were also generated using standardized values (Z-scores), which allowed for the comparison of the relative variations between the different parameters evaluated. Statistical analyses were performed using Statgraphics Centurion 19- X64 software, while multivariate analyses and graphical representations were performed in R version 4.5.2, using the FactoMineR, factoextra, tidyverse, and scales packages.

## 4. Conclusions

This study unravels the complex multi-factorial interaction between nitrogen forms, severe heat stress, and foliar proline application in determining the advanced physiological and metabolomic resilience of two contrasting tomato cultivars (Cherry and Tres Cantos). Biomass profiling revealed that a combined nitrogen regime (50/50) triggered severe ammonium toxicity, restricting vegetative growth and root development due to high metabolic assimilation costs, which completely masked potential proline-elicited shoot protection. Gas exchange analyses under varying light and CO_2_ curves demonstrated an unexpected biochemical photosynthetic acclimation at 43 °C governed by non-stomatal regulations in which enhanced carboxylation efficiency outpaced stomatal restrictions. Cultivar-specific water-use optimization split into a high-transpiration evaporative cooling strategy in Cherry versus conservative water retention in Tres Cantos. At the metabolomic level, the multi-factorial ANOVA confirmed that while core glutamate and aspartate pools remained homeostatically buffered, concurrent heat and ammonium stress induced a massive up-regulation of glutamine and asparagine as primary detoxification nitrogen sinks. Simultaneously, the biogenic amine profile shifted dramatically; concurrent stress stimulated high-order accumulation of putrescine and histamine through the activation of arginine-dependent decarboxylation pathways, with Cherry exhibiting significantly higher accumulation plasticity. Critically, foliar proline functioned as a precise metabolic modulator rather than a raw biomass promoter; it selectively rescued Cherry’s root biomass under heat, while globally stabilizing polyamine profiles by shifting low-molecular-weight diamines into highly protective triamine and tetramine forms (spermidine and spermine). Collectively, these findings demonstrate that heat resilience in tomato is not an isolated trait, but a synchronized response driven by nitrogen-form dependent metabolic channeling and proline-mediated homeostatic plasticity. Further studies including ROS accumulation and antioxidant enzyme activities are needed to elucidate the mechanisms underlying the protective effects of proline and nitrogen nutrition under heat stress.

## Figures and Tables

**Table 1:** Effect of two forms of nitrogen application (NO_3_^−^ and NO_3_^−^/NH_4_^+^) on two varieties of tomato plants (Cherry and Tres Cantos) with temperature changes and foliar application of proline: amino acid profiles. Lowercase letters indicate the differences between the tomato varieties, and different uppercase letters indicate the differences between the various temperatures and the foliar application of proline within the same variety (*p* ≤ 0.05, Duncan test, mean ± SE (*n* = 5)).

Amino Acid (µmol/g DW)	25 °C				43 °C				43 °C + Proline			
	(100/0) (NO_3_^−^)		(50/50) (NO_3_^−^/NH_4_^+^)		(100/0) (NO_3_^−^)		(50/50) (NO_3_^−^/NH_4_^+^)		(100/0) (NO_3_^−^)		(50/50) (NO_3_^−^/NH_4_^+^)	
	Cherry	Tres Cantos	Cherry	Tres Cantos	Cherry	Tres Cantos	Cherry	Tres Cantos	Cherry	Tres Cantos	Cherry	Tres Cantos
Histidine	0.62 ± 0.002 cD	0.66 ± 0.007 cC	1.62 ± 0.13 aC	0.96 ± 0.03 bBC	0.78 ± 0.01 dD	1.16 ± 0.15 cB	3.38 ± 0.13 aA	3.02 ± 0.10 bA	0.88 ± 0.03 bD	1.24 ± 0.08 bB	2.49 ± 0.43aB	2.92 ± 0.28 aA
Threonine	1.41 ± 0.40 aC	2.48 ± 0.48 aC	2.30 ± 0.21 aB	2.89 ± 0.79 aBC	3.18 ± 0.23 cB	4.05 ± 0.35 bcABC	5.40 ± 0.48 aA	4.73 ± 0.38 abAB	3.37 ± 0.20 bB	4.19 ± 0.24 abABC	3.98 ± 0.31 abB	5.14 ± 0.64 aA
Lysine	0.30 ± 0.09 cC	0.36 ± 0.06 cB	1.84 ± 0.13 aC	0.97 ± 0.23 bB	1.39 ± 0.27 bC	2.35 ± 0.70 bB	10.87 ± 1.18 aA	8.62 ± 0.87 aA	1.49 ± 0.33 bC	2.87 ± 0.68 bB	6.22 ± 0.89 aB	6.75 ± 1.45 aA
Methionine	0.006 ± 0.002 bC	0.02 ± 0.004 aB	0 ± 0 bC	0.006 ± 0.002 bB	0.06 ± 0.002 aA	0.09 ± 0.006 aA	0.03 ± 0.01 bBC	0.009 ± 0.004 bB	0.04 ± 0.004 bB	0.09 ± 0.003 aA	0.008 ± 0.006 cC	0.03 ± 0.01 bB
Valine	0 ± 0 bB	0 ± 0 bD	0.05 ± 0.03 abB	0.40 ± 0.23 aCD	1.27 ± 0.25 cB	1.91 ± 0.48 bcBC	4.16 ±1.14 abA	4.51 ± 0.86 aA	1.63 ± 0.52 aB	2.72 ± 0.38 aB	1.26 ± 0.45 aB	2.31 ± 0.85 aB
Isoleucine	0.22 ± 0.07 cD	0.29 ± 0.05 bcC	1.04 ± 0.19 aC	0.66 ± 0.16 abC	0.91 ± 0.08 bC	1.41 ± 0.21 bB	3.31 ± 0.28 aA	2.86 ± 0.01 aA	1.03 ± 0.13 cC	1.61 ± 0.16 bcB	2.61 ± 0.31 aB	2.35 ± 0.34 abA
Leucine	0.28 ± 0.08 cD	0.35 ± 0.06 bcC	1.20 ± 0.20 aC	0.76 ± 0.18 bC	1.03 ± 0.09 bC	1.58 ± 0.23 bB	3.68 ± 0.31 aA	3.17 ± 0.02 aA	1.15 ± 0.14 cC	1.79 ± 0.17 bcB	2.93 ± 0.35 aB	2.62 ± 0.37 abA
Phenylalanine	0.52 ± 0.11 bD	0.56 ± 0.08 bD	1.07 ± 0.14 aC	0.76 ± 0.15 abD	1.01 ± 0.08 bCD	1.07 ± 0.13 bCD	2.80 ± 0.27 aA	2.58 ± 0.03 aA	1.20 ± 0.08 bC	1.35 ± 0.12 abC	1.96 ± 0.28 aB	1.84 ± 0.25 abB
Tryptophan	0.14 ± 0.03 cD	0.31 ± 0.04 bcC	1.50 ± 0.33 aB	0.81 ± 0.11 bC	0.59 ± 0.05 cCD	1.95 ± 0.31 bB	3.75 ± 0.10 aA	3.77 ± 0.19 aA	0.84 ± 0.07 cC	2.04 ± 0.16 bB	3.34 ± 0.32 aB	3.51 ± 0.45 aA
Arginine	0.19 ± 0.02 bC	0.26 ± 0.01 bcB	0.97 ± 0.21 aC	0.62 ± 0.15 abB	0.29 ± 0.03 bC	0.48 ± 0.14 bB	5.47 ± 0.93 aA	4.32 ± 0.75 aA	0.42 ± 0.08 cC	1.09 ± 0.35 cB	3.15 ± 0.28 bB	4.85 ± 0.55 aA
Asparagine	0.60 ± 0.17 bD	1.05 ± 0.18 bB	6.73 ± 1.57 aC	6.13 ± 2.13 aB	2.16 ± 0.12 bCD	4.63 ± 0.64 bB	18.26 ± 1.96 aA	21.71 ± 3.47 aA	2.83 ± 0.59 cCD	5.21 ± 0.35 cB	13.08 ± 2.16 bB	23.65 ± 1.84 aA
Serine	1.55 ± 0.36 bB	2.23 ± 0.36 bB	4.85 ± 1.20 aA	3.24 ± 0.58 abB	3.01 ± 0.16 aAB	3.23 ± 0.21 aB	6.20 ± 1.61 aA	6.88 ± 1.90 aB	4.09 ± 0.79 bAB	4.48 ± 0.16 bB	4.81 ± 0.84 bAB	11.84 ± 1.55 aA
Glycine	0.12 ± 0.04 bD	0.19 ± 0.04 abB	0.56 ± 0.17 aBCD	0.48 ± 0.15 abB	0.40 ± 0.0003 bCD	0.40 ± 0.0001 bB	1.24 ± 0.19 aA	0.99 ± 0.19 aA	0.81 ± 0.28 abABC	0.56 ± 0.04 bB	1.05 ± 0.03 abAB	1.23 ± 0.15 aA
Glutamine	8.52 ± 2.30 bC	11.26 ± 1.95 bC	36.89 ± 6.48 aB	27.48 ± 5.63 aB	21.16 ± 0.53 bBC	24.20 ± 1.78 bBC	85.75 ± 8.96 aA	72.15 ± 6.04 aA	27.90 ± 6.57 bBC	32.86 ± 1.24 bB	69.82 ± 5.56 aA	63.02 ± 4.02 aA
L-alanine	2.74 ± 0.66 bB	4.72 ± 0.75 abC	6.69 ± 1.97 aAB	5.11 ± 0.98 abC	11.21 ± 0.88 aA	10.50 ± 0.95 aB	12.23 ± 1.63 aA	11.19 ± 0.96 aB	9.44 ± 1.43 bA	10.25 ± 1.11 bB	10.69 ± 1.36 bA	15.14 ± 1.10 aA
Proline	0.71 ± 0.17 bC	0.64 ± 0.11 bB	5.04 ± 1.26 aB	1.35 ± 0.31 bB	0.97 ± 0.08 bC	0.86 ± 0.04 bB	2.15 ± 0.31 aC	1.60 ± 0.15 aB	6.03 ± 0.38 aB	7.79 ± 0.47 aA	8.50 ± 1.10 aA	6.47 ± 1.01 aA
Cysteine	0.0006 ± 0.0004 bB	0 ± 0 bC	0.03 ± 0.01 aA	0.02 ± 0.007 abA	0.002 ± 0.001 aB	0.002 ± 0.001 aBC	0.005 ± 0.002 aB	0.003 ± 0.001 aBC	0.006 ± 0.002 abB	0.01 ± 0.005 aB	0 ± 0 bB	0.002 ± 0.002 bBC
Tyrosine	0.27 ± 0.07 bD	0.28 ± 0.02 bB	1.23 ± 0.25 aC	0.66 ± 0.13 bB	0.81 ± 0.24 bCD	1.12 ± 0.34 bB	4.63 ± 0.44 aA	3.69 ± 0.37 aA	1.18 ± 0.29 bCD	1.19 ± 0.25 bB	3.25 ± 0.26 aB	3.01 ± 0.45 aA
Aspartic acid	4.42 ± 0.77 aC	4.97 ± 1.19 aC	5.66 ± 1.37 aBC	8.01 ± 1.67 aABC	13.90 ± 1.17 aA	11.57 ± 1.02 aAB	8.43 ± 0.59 bB	6.66 ± 0.74 bBC	12.95 ± 0.85 aA	13.48 ± 0.27 aA	8.75 ± 0.70 aB	12.71 ± 3.12 aA
Glutamic acid	7.19 ± 1.47 aB	8.58 ± 1.37 aB	7.04 ± 1.68 aB	6.18 ± 0.47 aB	15.85 ± 0.84 aA	16.41 ± 0.25 aA	8.03 ± 1.86 bB	9.02 ± 0.43 bB	15.03 ± 0.11 aA	17.97 ± 1.29 aA	7.07 ± 0.68 bB	14.67 ± 2.99 aA
Hydroxyproline	0.097 ± 0.03 bC	0.12 ± 0.01 bC	0.31 ± 0.03 aAB	0.09 ± 0.006 bC	0.19 ± 0.003 cBC	0.17 ± 0.01 cC	0.37 ± 0.009 aA	0.26 ± 0.01 bB	0.16 ± 0.009 a BC	0.17 ± 0.009 aC	0.26 ± 0.12 aABC	0.37 ± 0.04 aA
All-isoleucine	0.26 ± 0.07 cC	0.35 ± 0.04 cC	1.09 ± 0.13 aB	0.68 ± 0.15 bBC	0.55 ± 0.07 bBC	0.73 ± 0.14 bBC	2.29 ± 0.34 aA	2.16 ± 0.17 aA	0.76 ± 0.14 bBC	1.01 ± 0.12 abB	1.82 ± 0.38 aA	1.76 ± 0.36 aA
Ethanolamine	2.07 ± 0.41 bB	2.33 ± 0.39 bB	3.73 ± 0.45 abA	4.08 ± 0.81 aA	4.31 ± 0.28 aAB	4.19 ± 0.32 aAB	4.97 ± 0.83 aA	4.82 ± 0.29 aA	4.38 ± 0.37 aAB	5.66 ± 0.72 aA	5.09 ± 0.35 aA	5.48 ± 0.65 aA
Citrulline	0.19 ± 0.05 aA	0.30 ± 0.06 aAB	0.14 ± 0.03 aA	0.34 ± 0.09 aAB	0.25 ± 0.04 aA	0.24 ± 0.02 abB	0.13 ± 0.008 bA	0.22 ± 0.04 abB	0.20 ± 0.02 bA	0.42 ± 0.08 aAB	0.11 ± 0.02 bA	0.45 ± 0.04 aA
Alpha-aminoadipic acid	13.73 ± 1.84 aA	13.07 ± 2.11 aB	13.30 ± 2.20 aA	9.96 ± 0.67 Ab	17.56 ± 1.00 aA	18.51 ± 1.60 aAB	18.41 ± 1.22 aA	15.16 ± 0.22 aB	18.38 ± 1.16 aA	21.98 ± 0.83 aA	17.27 ± 1.74 aA	19.27 ± 2.96 aAB
Beta-aminoisobutyric acid	0.06 ± 0.007 bB	0.10 ± 0.02 bC	0.05 ± 0.01 bAB	0.18 ± 0.02 aA	0.12 ± 0.001 aAB	0.15 ± 0.01 aAB	0.05 ± 0.01 bB	0.12 ± 0.01 aBC	0.14 ± 0.01 aA	0.16 ± 0.006 aAB	0.06 ± 0.03 bB	0.15 ± 0.02 aAB
Ornithine	0 ± 0 aA	0.004 ± 0.002 aA	0.01 ± 0.008 aA	0.02 ± 0.01 aA	0 ± 0 bA	0.01 ± 0.007 bA	0.12 ± 0.04 aA	0.11 ± 0.03 aA	0.35 ± 0.25 aA	0.02 ± 0.01 aA	0.02 ± 0.01 aA	0.14 ± 0.05 aA
Homocysteine	0.04 ± 0.004 cAB	0.08 ± 0.01 bAB	0.03 ± 0.009 cA	0.12 ± 0.003 aA	0.05 ± 0.004 bAB	0.09 ± 0.006 aAB	0.02 ± 0.01 bAB	0.05 ± 0.01 bC	0.05 ± 0.007 bAB	0.11 ± 0.01 aAB	0.01 ± 0.009 cB	0.08 ± 0.006 abBC
Total	46.28 ± 8.17 bC	55.62 ± 8.23 bC	105.06 ± 15.58 aB	83.25 ± 14.73 abBC	104.11 ± 4.32 bB	114.29 ± 6.79 bB	216.38 ± 17.09 aA	194.73 ± 11.36 aA	115.93 ± 8.38 bB	122.84 ± 14.49 bB	179.65 ± 11.08 aA	196.97 ± 14.92 aA

**Table 2:** ANOVA summary of amino acids responses to nitrogen form (NO_3_^−^/NH_4_^+^), heat stress (43 °C), and exogenous proline in two tomato varieties. Analysis of variance: ns, not significant; * *p* ≤ 0.05; ** *p* ≤ 0.005; *** *p* ≤ 0.001.

Amino Acid (µmol/g DW)	25 °C			43 °C			43 °C + Proline		
	A: Variety	B: NO_3_^−^/NH_4_^+^	A × B	A: Variety	B: NO_3_^−^/NH_4_^+^	A × B	A: Variety	B: NO_3_^−^/NH_4_^+^	A × B
Histidine	***	***	***	***	***	***	***	***	***
Threonine	ns	ns	ns	ns	ns	ns	ns	ns	ns
Lysine	*	***	*	*	***	*	*	***	*
Methionine	*	*	ns	*	*	ns	*	*	ns
Valine	ns	ns	ns	ns	ns	ns	ns	ns	ns
Isoleucine	ns	***	ns	ns	***	ns	ns	***	ns
Leucine	ns	***	ns	ns	***	ns	ns	***	ns
Phenylalanine	ns	*	ns	ns	*	ns	ns	*	ns
Tryptophan	ns	***	*	ns	***	*	ns	***	*
Arginine	ns	***	ns	ns	***	ns	ns	***	ns
Asparagine	ns	***	ns	ns	***	ns	ns	***	ns
Serine	ns	*	ns	ns	*	ns	ns	*	ns
Glycine	ns	*	ns	ns	*	ns	ns	*	ns
Glutamine	ns	***	ns	ns	***	ns	ns	***	ns
L-alanine	ns	ns	ns	ns	ns	ns	ns	ns	ns
Proline	*	**	*	*	**	*	*	**	*
Cysteine	ns	**	ns	ns	**	ns	ns	**	ns
Tyrosine	ns	***	ns	ns	***	ns	ns	***	ns
Aspartic acid	ns	ns	ns	ns	ns	ns	ns	ns	ns
Glutamic acid	ns	ns	ns	ns	ns	ns	ns	ns	ns
Hydroxyproline	***	**	***	***	**	***	***	**	***
All-isoleucine	ns	***	*	ns	***	*	ns	***	*
Ethanolamine	ns	*	ns	ns	*	ns	ns	*	ns
Citrulline	*	ns	ns	*	ns	ns	*	ns	ns
Alpha-aminoadipic acid	ns	ns	ns	ns	ns	ns	ns	ns	ns
Beta-aminoisobutyric acid	***	ns	*	***	ns	*	***	ns	*
Ornithine	ns	ns	ns	ns	ns	ns	ns	ns	ns
Homocysteine	***	ns	*	***	ns	*	***	ns	*
Total	ns	*	ns	ns	*	ns	ns	*	ns

**Table 3:** Effect of two forms of nitrogen application (NO_3_^−^ and NO_3_^−^/NH_4_^+^) on two varieties of tomato plants (Cherry and Tres Cantos) with temperature changes and foliar application of proline: polyamines profiles. Lowercase letters indicate the differences between the tomato varieties, and different uppercase letters indicate the differences between the various temperatures and the foliar application of proline (*p* ≤ 0.05, Duncan test, mean ± SE (n = 5)).

Polyamines (µmol/g DW)	25 °C				43 °C				43 °C + Proline			
	(100/0) (NO_3_^−^)		(50/50) (NO_3_^−^/NH_4_^+^)		(100/0) (NO_3_^−^)		(50/50) (NO_3_^−^/NH_4_^+^)		(100/0) (NO_3_^−^)		(50/50) (NO_3_^−^/NH_4_^+^)	
	Cherry	Tres Cantos	Cherry	Tres Cantos	Cherry	Tres Cantos	Cherry	Tres Cantos	Cherry	Tres Cantos	Cherry	Tres Cantos
Putrescine	0.44 ± 0.09 bC	0.35 ± 0.04 bD	2.10 ± 0.39 aB	1.01 ± 0.24 bC	0.45 ± 0.07 cC	0.55 ± 0.07 cD	4.33 ± 0.19 aA	2.65 ± 0.27 bA	0.44 ± 0.02 bC	0.75 ± 0.06 bCD	2.46 ± 0.60 aB	1.99 ± 0.13 aB
Histamine	0.04 ± 0.02 bC	0.08 ± 0.01 bB	0.20 ± 0.01 abBC	0.29 ± 0.10 aB	0.13 ± 0.009 bC	0.21 ± 0.03 bB	0.90 ± 0.13 aA	1.39 ± 0.31 aA	0.16 ± 0.02 cC	0.26 ± 0.01 bcB	0.52 ± 0.15 bB	1.23 ± 0.08 aA
Cadaverine	0.39 ± 0.30 aA	0 ± 0 aA	0.11 ± 0.07 aA	0.06 ± 0.04 aA	0.83 ± 0.17 aA	0 ± 0 aA	0.79 ± 0.44 aA	0.60 ± 0.24 aA	0.93 ± 0.31 aA	0.29 ± 0.20 aA	0 ± 0 aA	1.16 ± 1.10 aA
Spermidine	0.64 ± 0.18 aBC	0.59 ± 0.15 aB	0.90 ± 0.15 aAB	0.82 ± 0.28 aAB	0.93 ± 0.06 bAB	0.83 ± 0.03 bB	1.19 ± 0.009 aA	0.98 ± 0.08 bAB	0.97 ± 0.07 bAB	1.43 ± 0.11 aA	0.56 ± 0.04 cC	0.83 ± 0.20 bcB
Spermine	0.19 ± 0.02 bD	0.25 ± 0.03 abD	0.40 ± 0.11 aC	0.31 ± 0.03 abCD	0.58 ± 0.01 abA	0.74 ± 0.06 aB	0.54 ± 0.01 bAB	0.65 ± 0.09 abBC	0.60 ± 0.04 bA	1.02 ± 0.14 aA	0.45 ± 0.03 bBC	0.68 ± 0.08 bBC
Total	1.72 ± 0.58 bB	1.27 ± 0.21 bC	3.71 ± 0.56 aB	2.49 ± 0.66 abBC	2.80 ± 0.13 cB	2.33 ± 0.15 cC	7.75 ± 0.50 aA	6.28 ± 0.65 bA	3.10 ± 0.36 bB	3.69 ± 0.44 abABC	3.98 ± 0.71 abB	5.53 ± 1.09 aAB

**Table 4:** ANOVA Summary of Polyamines Responses to Nitrogen Form (NO_3_^−^/NH_4_^+^), Heat Stress (43 °C), and Exogenous Proline in Two Tomato Varieties. Analysis of Variance: ns, Not Significant; * *p* ≤ 0.05; ** *p* ≤ 0.005; *** *p* ≤ 0.001.

Polyamines (µmol/g DW)	25 °C			43 °C			43 °C + Proline		
	A: Variety	B: NO_3_^−^/NH_4_^+^	A × B	A: Variety	B: NO_3_^−^/NH_4_^+^	A × B	A: Variety	B: NO_3_^−^/NH_4_^+^	A × B
Putrescine	*	***	ns	***	***	***	ns	***	ns
Histamine	ns	**	ns	ns	***	ns	***	***	*
Cadaverine	ns	ns	ns	ns	ns	ns	ns	ns	ns
Spermidine	ns	ns	ns	*	**	ns	*	**	ns
Spermine	ns	*	ns	*	ns	ns	**	*	ns
Total	ns	*	ns	*	***	ns	ns	ns	ns

## Data Availability

The original contributions presented in this study are included in the article. Further inquiries can be directed to the corresponding authors.
